# Genetic association of -1562C>T polymorphism in the *MMP9* gene with primary glaucoma in a north Indian population

**DOI:** 10.1371/journal.pone.0192636

**Published:** 2018-02-12

**Authors:** Nanamika Thakur, Manu Kupani, Rajeev Kumar Pandey, Rashim Mannan, Archna Pruthi, Sanjana Mehrotra

**Affiliations:** 1 Department of Human Genetics, Guru Nanak Dev University, Amritsar, Punjab, India; 2 Johns Hopkins Bloomberg School of Public Health, Baltimore, Maryland, United States of America; 3 Baba Deep Singh Charitable Hospital, Amritsar, Punjab, India; Oregon Health and Science University, UNITED STATES

## Abstract

MMP (Matrix metalloproteinase) 9 is reported to affect glaucoma pathogenesis by altering intraocular pressure (IOP) through its role in remodeling the extracellular matrix (ECM) in the trabecular meshwork. A genetic variant at the promoter region in the *MMP9* gene (-1562C>T) has a putative role in regulating its transcription rate and hence can affect genetic predisposition to primary glaucoma. The present study examined the association of -1562C>T promoter polymorphism in the *MMP9* gene with Primary Open Angle Glaucoma (POAG) and Primary Angle Closure Glaucoma (PACG) in a north Indian population. A total of 729 subjects (POAG = 224, PACG = 138 and 367 controls) were recruited for the study. Genotyping for the promoter sequence variant was done with PCR-RFLP method. Genotypic and allelic frequency distribution of the POAG and PACG data sets were compared to that of controls by chi-square test and genetic association was tested under different genetic models as implemented under PLINK. Statistically significant difference was observed in the genotype frequencies between PACG cases and controls (p = 0.030). However, in the POAG cases, this difference was only borderline (p = 0.052). Genetic model analysis, under the dominant model revealed 1.6 and 1.4 fold increased susceptibility to PACG and POAG (p = 0.012, p = 0.032) respectively. A higher frequency of CT genotype was observed in PACG as well as POAG males as compared to female subjects. According to the dominant model, CT+TT genotype conferred 1.8 fold higher risk of developing PACG among male patients as compared to the control group (p = 0.048, OR = 1.87;1.00–3.50). Current findings suggest significant association of *MMP9* -1562C>T polymorphism with primary glaucoma in the targeted north Indian population and warrant further replication of the findings in other populations.

## Introduction

Glaucoma, an optic neuropathy is the second leading cause of blindness worldwide after cataract [1, 2]. Being a complex disorder, it manifests in different clinical forms, among which primary open angle and angle closure account for bulk of the cases [3–6]. The vision loss occurs due to gradual damage to the retinal ganglion cells (RGCs) and the optic nerve in response to elevated intraocular pressure (IOP) which is a major risk factor for glaucoma development [7]. Genetics is another important risk factor as suggested by higher risk of both open angle and angle closure glaucoma among first-degree relatives of affected individuals in twin and family based studies [8–10]. Several genetic association studies have identified loci which might influence the genetic predisposition to glaucoma development and progression [9, 10], yet these variants explain only less than 10% of heritability [9]. The progressive and irreversible apoptosis of RGCs, the axons of which form the optic nerve is an important pathogenic feature in glaucoma [11]. The primary site of damage in glaucoma is controversial, it could be the optic nerve head (ONH) or retina, in either case elevation in IOP is an important contributing factor [11]. The initiating molecular events leading to high IOP conditions in the eye are not completely understood, but may occur primarily due to an imbalance of aqueous humor production by the ciliary body and its outflow resistance via the trabecular meshwork (TM) [12]. Since the TM determines the outflow resistance by homeostatic turnover of its extracellular matrix (ECM), pathways/proteins affecting ECM remodeling assume importance in glaucoma pathogenesis and can be targets for therapeutic intervention [13]. Matrix metalloproteinases (MMPs), a group of zinc proteinases are involved in degradation of ECM at TM and lamina cribosa (LC) [14]. Among different MMPs, *MMP9* encodes a 92-kDa multidomain zinc dependent enzyme known as gelatinase or type V collagenase and is known to extensively affect ECM deposition and turnover in the TM and LC regions in glaucoma [14]. Numerous studies have linked changes in the expression of *MMP9* in the retina, optic nerve, aqueous humor, and TM with glaucomatous eyes in humans [15–17] and animal models of glaucoma [18–19]. The altered expression of MMPs could be a response to elevated IOP and simultaneously contribute to it by changing the outflow resistance. In addition to their defining role in affecting IOP by remodeling of ECM of the TM in the anterior segment of the eye [20], abnormal expression of *MMP9* can also affect RGCs survival as shown by Guo et alwhere MMP9 levels correlated with elevation in IOP and RGC apoptosis [7]. Aberrant MMP9 activity has also been implicated in both ischemia and excitotoxicity-mediated RGC damage [21]. In ischemic conditions wherein membrane depolarization is one of the initiating events for injury, injection of depolarizing agents like KCl into vitreous humor of mice induces up-regulation of Mmp9 activity in the retina [21]. This depolarization-induced Mmp9 up-regulation is through N-methyl-D-aspartate (NMDA) and non-NMDA type glutamate receptors as intravitreal injection of glutamate receptor antagonists along with KCl, resulted in reduced Mmp9 activity [21–22]. In another study, NMDA mediated excitotoxic damage to RGCs was shown to be through Mmp9 activation via neuronal nitric oxide synthase [23]. The molecular mechanisms underlying how MMP9 contributes to RGC death are not clearly understood but may involve their role in degradation of laminin, one of the major components of the basement membrane on the inner wall of Schlemm’s canal. Experiments with Mmp9 deficient mice have indicated a direct causal relationship between MMP9 and degradation of laminin [24–25]. Genetic variants in the *MMP9* gene, specifically cis-regulatory elements which provide a binding site for transcription factors, can influence its expression and hence may modify overall genetic risk for glaucoma onset or progression [26]. rs3918242 (-1562C>T) variant in the promoter region exerts a functional effect on the transcription of *MMP9* gene and has been found to be associated with high MMP9 levels in serum samples [27]. The present study therefore aims to assess whether -1562C>T polymorphism in the *MMP9* gene promoter is associated with primary glaucoma in a north Indian population since the polymorphism has not been investigated in the targeted population, more so in any Indian population.

## Materials and methods

### Study participants

Study participants were recruited from Baba Deep Singh Eye hospital, Amritsar (Punjab), India after obtaining written informed consent. The study was approved by the Research Ethical Committee of Guru Nanak Dev University, Amritsar, Punjab, India and the study protocols were in accordance to the principles of Declaration of Helsinki. 362 primary glaucoma patients (POAG = 224 and PACG = 138) were enrolled. For POAG patients, the inclusion was based on following criteria: IOP of greater than 21mm Hg in either of the eyes tested using Goldmann Applanation Tonometry, glaucomatous ONH damage defined as a vertical cup-disc ratio (VCDR) 0.7 or greater as adjudged clinically on slit lamp biomicroscopy using hand held +90 D. This was confirmed using contrast enhanced fundus photograph on optical coherence tomography (OCT) as well as optic disc analysis or glaucomatous visual field defect as detected on automated perimeter using Humpherys Visual Field Analyser using Swedish Interactive Thresholding Algorithm (SITA) standard protocols. PACG cases were also recruited on the basis of above described criteria along with the presence of at least 180 degrees of closed angle in which the TM is not visible on gonioscopy. The control data set consisted of 367 unrelated age and gender matched subjects without any family history of glaucoma. The controls were all examined, prior to cataract surgery, for IOP less than 21 mmHg, normal visual field, normal optic nerve heads with CDR of <0.5. Individuals with known chronic systemic inflammatory diabetes (RBS-140mg/Dl; according to American Diabetes Association (ADA) guidelines), autoimmune or immunosuppressive disease as well as a pre-existing ocular disease (diabetic retinopathy, age-related macular degeneration) were excluded from the study. Individuals having ocular hypertension (OHT) were also excluded from the study group.

### Sample collection, DNA isolation and Genotyping

**Fig 1:**
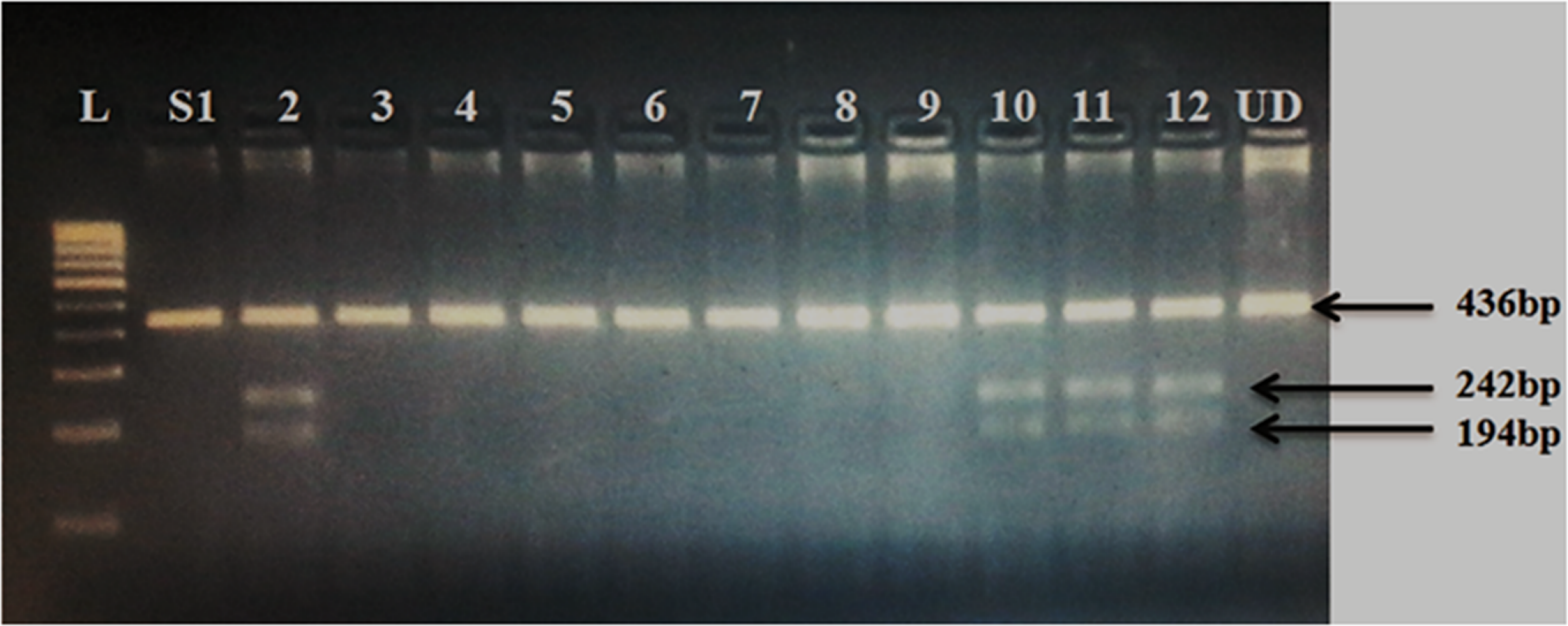
Digested PCR product of *MMP9* -1562C>T. Well1 = 100bp Ladder; S2,10,11,12 = Heterozygous (436/242/194bp); S1,3,4,5,6,7,8,9 = Homozygous wild (436bp); UD = Undigested PCR product.

**Fig 2:**
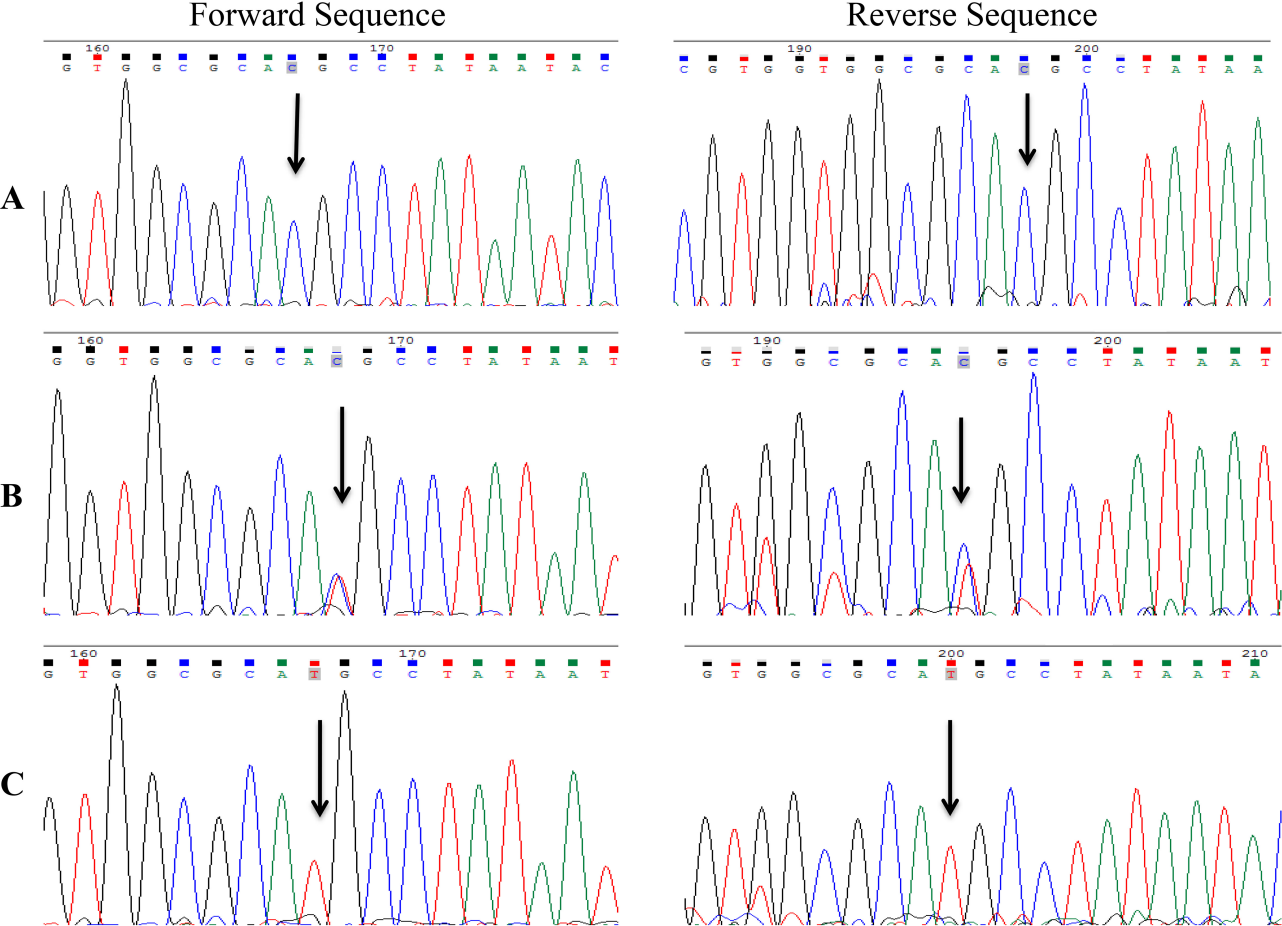
Sanger sequencing chromatograms for validation of genotypes. A) Homozygous CC genotype B) Heterozygous CT genotype C) Homozygous TT genotype.

Venous blood was collected in EDTA vials and genomic DNA was extracted using phenol chloroform method [28]. All samples were diluted to 100ng/μl stock concentration. Quantification of extracted DNA was done using NanoDrop ND-2000 spectrophotometer (NanoDrop Technologies, Wilmington, DE, USA). The target region (436bp) was amplified with the primer sequences and conditions as previously reported [29]. PCR was performed in a final reaction volume of 15μl containing 50ng DNA, 1X Taq Polymerase buffer reagent, 2.5mmol of each deoxynucleotide triphosphate (dNTPs) (3B BlackBio Biotech India Ltd), 20mmol MgCl_2_, 0.3μmol/μl of each primer, and 0.09 units Taq DNA polymerase (3B BlackBio Biotech India Ltd; 5U/μl). The cycling conditions were initial denaturation at 95°C for 10 min, followed by 40 cycles at 95°C for 45 s, 61.5°C for 45 s and 72°C for 45 s, with a final extension at 72°C for 10 min. After amplification, the PCR products were digested with 3 units of Sph-I (New England Biolabs) for 12h at 37°C. The digested products were separated by electrophoresis on 2.5% agarose gel stained with ethidium bromide (10mg/ml) (GeNei^TM^) and genotypes were scored on the basis of Restriction Fragment Length Polymorphism (RFLP) pattern as given in Fig 1. As a quality control measure, genotypes in few samples were confirmed by sanger sequencing (Fig 2).

### Statistical analysis

Descriptive statistical analysis for demographic and clinical characteristics for study participants was performed using GraphPad Prism 5.0. The results were tabulated as mean ± standard deviation (SD). Association analyses were performed using PLINK software (v1.07). Odds ratios (ORs) for various genetic models, dominant, recessive, additive and allelic were calculated by Medcalc software. P-values of less than 0.05 were considered to be statistically significant. To investigate the association of the variant with IOP and VCDR which are known endophenotypes of glaucoma, the values for right eye was chosen arbitrarily (as the mean for both eyes was same, Table 1) for ANOVA.

**Table 1:** Clinical and demographic details of the study groups. Values are expressed as mean ± SD.

Variable		POAG Cases (n = 224)	PACG Cases (n = 138)	Control (n = 367)
**Age**		59.1 ± 13.3	61.6 ± 10.4	58.1 ± 13.3
**IOP**	Right	21.6 ± 8.91	23.7 ± 9.49	14.7 ± 3.44
	Left	21.4 ± 8.50	23.2 ± 8.91	14.6 ± 3.40
**CD**	Right	0.75 ± 0.55	0.70 ± 0.16	0.23 ± 0.09
	Left	0.74 ± 0.55	0.72 ± 0.15	0.23 ± 0.09

**IOP** = Intraocular pressure, **CD** = Cup to disc ratio

## Results

The present case control genetic association study was conducted on 729 individuals, comprising 224 POAG cases, 138 PACG cases and 367 control subjects. The demographic and clinical parameters of these groups are presented in Table 1.

**Fig 3:**
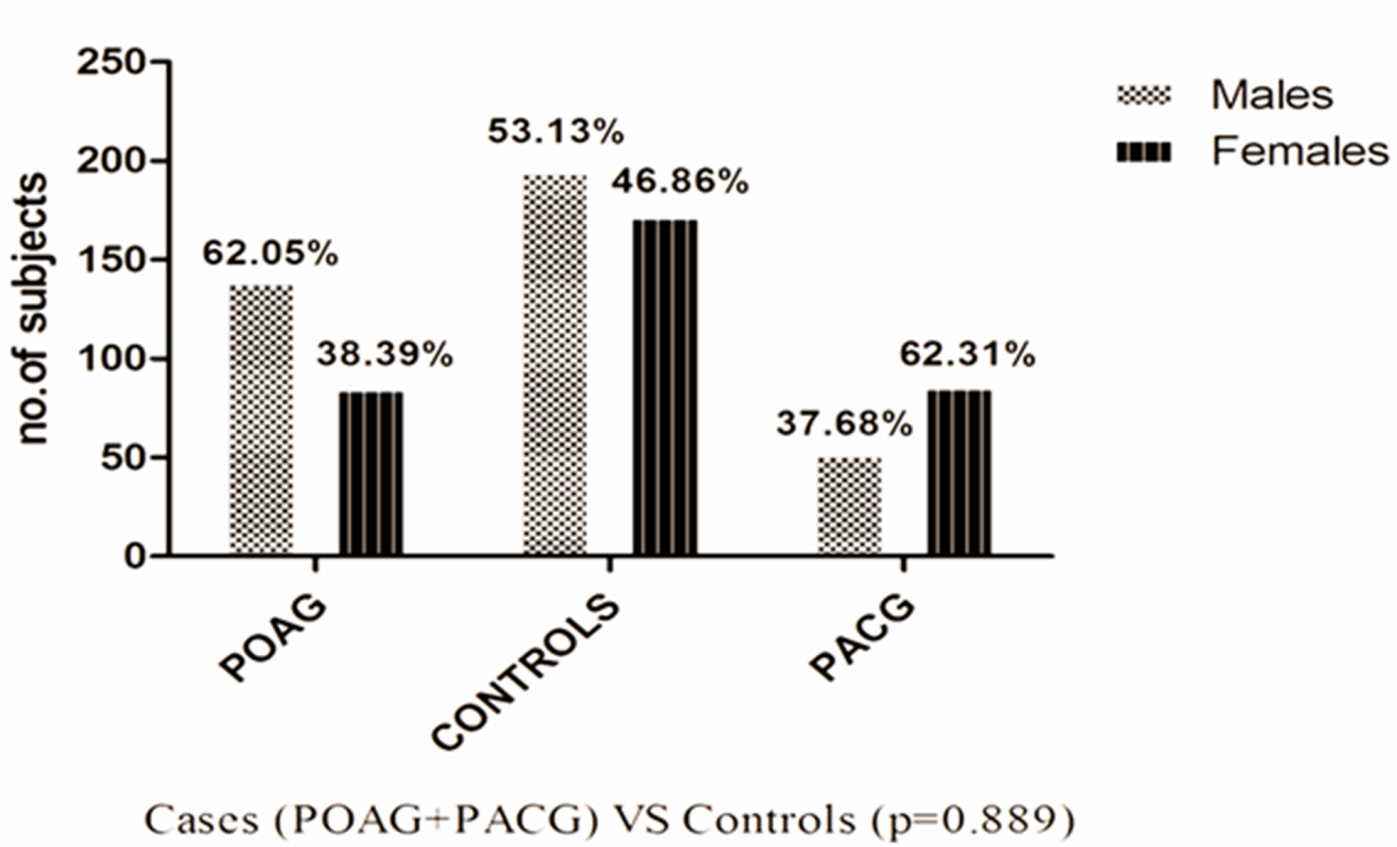
Frequency distribution of males and females among POAG and PACG cases with respect to control subjects.

Frequency of males (62.05%) among POAG cases was higher as compared to PACG (37.68%), while the female subjects were more prevalent in PACG (62.31%) with respect to POAG (38.39%). These results highlight the gender difference in glaucoma prevalence; women are known to be at a higher risk for PACG [30]. Although the gender predilection for POAG is not very clear, in the present study prevalence of POAG was found to be higher in men [30]. Total number of males and females in cases (both PAOG/PACG) and controls did not show any significant difference (p = 0.889) as given in Fig 3.

### -1562C>T *MMP9* polymorphism revealed significant association with PACG

The observed genotypes for rs3918242 followed Hardy Weinberg equilibrium (HWE) in controls (observed and expected heterozygosity = 0.28, 0.26 respectively; p = 0.244). The genotype and allele frequency distribution along with genetic model analysis for subjects of PACG and controls is given in Table 2. PACG cases showed statistically significant difference in allelic and genotypic frequencies with respect to controls. 22.82% PACG cases had minor allele, ‘T’ whereas 15.94% control individuals had T allele (p = 0.011), thereby making it the risk allele in our population. The frequencies of CC, CT and TT genotypes were 57.97%, 38.40%, 3.62% in PACG patients while in controls the corresponding frequencies were 69.75%, 28.61%, 1.63% (p = 0.030). Analysis of genotypic distribution revealed ~1.6 fold increased susceptibility to PACG under dominant (CT+TT vs CC) (p = 0.012, OR = 1.65; 1.11–2.50) and co-dominant models (CT vs CC+TT) (p = 0.035, OR = 1.55; 1.03–2.34) as shown in Table 2.

**Table 2:** Distribution of allele frequency, genotype frequency and genetic model of rs3918242 among PACG cases and controls.

rs3918242	PACG n (%)	Controls n (%)	p-value	OR (CI)
**Allele**			0.011*	1.53 (1.10–2.19)
C	213 (77.17)	617 (84.05)		
T	63 (22.82)	117 (15.94)		
**Genotype**				
**CC**	80 (57.97)	256 (69.75)	0.030*	
**CT**	53 (38.40)	105 (28.61)		
**TT**	5 (3.62)	6 (1.63)		
**Genetic Model**				
**Dominant** (CT+TT vs.CC)	58/80	111/256	0.012*	1.67 (1.11–2.50)
**Recessive** (TT vs. CT+CC	5/133	6/361	0.172	2.26 (0.67–7.53)
**Co-dominant** (CT vs. CC+TT)	53/85	105/262	0.035*	1.55 (1.03–2.34)
**Additive** (TT vs.CC)	5/80	6/256	0.113	2.66 (0.79–8.97)

* Indicates significant p-value

OR = Odds Ratio, CI = Confidence Interval

### Higher prevalence of CT genotype in PACG male patients

Subsequent segregation of PACG cases on the basis of gender (Table 3) revealed a different frequency distribution of genotypes among PACG males and females. A higher number of heterozygotes were observed in PACG males (40.38%) than in PACG females (34.88%) but the difference was not statistically significant. When the comparison of PACG males and females was done with healthy male and female subjects, the study failed to obtain any significant difference in the genotypic frequencies. Genetic model investigation did not reveal a statistically significant difference in genotypic distribution among affected and control female subjects. In contrast to females, the dominant model unveiled 1.8 fold higher risk towards PACG in males (p = 0.048, OR = 1.87; 1.00–3.05).

**Table 3:** Gender wise comparison of PACG cases and controls.

**Females with PACG vs. control females**				
Genotypes	PACG females n = 86 (%)	Control females n = 172 (%)	p-value	OR(CI)
CC	53(61.62)	124(72.09)	0.149	
CT	30(34.88)	46(26.74)		
TT	3(3.48)	2(1.16)		
**Genetic Models**				
Dominant (CT+TT vs.CC)	33/53	48/124	0.087	1.61(0.93–2.78)
Recessive (TT vs. CT+CC)	3/83	2/170	0.201	3.07(0.50–18.8)
**Males with PACG vs. control males**				
Genotypes	PACG males n = 52(%)	Control males n = 195(%)	p-value	OR(CI)
CC	29(55.76)	137(70.25)	0.135	
CT	21(40.38)	54(27.69)		
TT	2(3.48)	4(2.05)		
**Genetic Models**				
Dominant (CT+TT vs.CC)	23/29	58/137	0.048^*^	1.87(1.00–3.50)
Recessive (TT vs. CT+CC)	2/50	4/191	0.455	1.91(0.34–10.7)

* Indicates significant p-value

OR = Odds Ratio, CI = Confidence Interval

### -1562C>T *MMP9* polymorphism revealed borderline significance with POAG

The prevalence of CC genotype was greater in controls (69.75%) than in cases (61.16%) while frequency of CT genotype was found to be higher in cases (37.94%) as compared to the control group (28.61%). Incidence of TT genotype was lesser in both POAG cases and controls (0.89%, 1.63% respectively). The difference in genotypic distribution showed only borderline significance (p = 0.052). The prevalence of minor allele ‘T’ was higher in cases (19.86%) than controls (15.94%), however the difference was not statistically significant. The genetic model analysis showed 1.5 fold increased susceptibility toward POAG, conferred by CT genotype under co-dominant model (p = 0.018, OR = 1.52; CI = 1.03–2.17). Dominant model also revealed a statistically different genotypic distribution between cases and controls (p = 0.032, OR = 1.46; CI = 1.07–2.17) (Table 4).

**Table 4:** Distribution of allele frequency, genotype frequency and genetic model of rs3918242 among POAG cases and controls.

rs3918242	POAG n (%)	Controls n (%)	p-value	OR (CI)
**Allele**			0.084	1.30(0.96–1.77)
C	359 (80.13)	617 (84.05)		
T	89 (19.86)	117 (15.94)		
**Genotype**				
CC	137 (61.16)	256 (69.75)	0.052*	
CT	85 (37.94)	105 (28.61)		
TT	2 (0.89)	6 (1.63)		
**Genetic Model**				
**Dominant** (CT+TT vs.CC)	87/137	111/256	0.032*	1.46(1.03–2.07)
**Recessive** (TT vs. CT+CC)	2/222	6/361	0.455	0.54(0.10–2.70)
**Co-dominant** (CT vs. CC+TT)	85/139	105/262	0.018*	1.52(1.07–2.17)
**Additive** (TT vs. CC)	2/137	6/256	0.565	0.62 (0.12–3.12)

* Indicates significant p-value

OR = Odds Ratio, CI = Confidence Interval

### Higher distribution of CT Genotype among POAG males than female patients

PAOG cases were segregated into two sub-groups of males and females. The frequency of CT genotype was found to be higher in affected males (37.41%) with respect to affected females (34.11%), while CC genotype was observed to be more in number among female patients as compared to males having POAG (64.70%, 62.58%). None of the POAG males had TT genotype in contrast to control males (2.05%) as shown in Table 5. The difference in genotypic distribution between POAG males and control male subjects was close to significance threshold (p = 0.050). However, the affected females did not show any difference in genotypic frequency as compared to control females. Genetic model analysis failed to obtain any difference in genotypic distribution among male and female patients with respect to corresponding controls. No significant correlation was observed between -1562C>T polymorphism and IOP and VCDR in POAG and PACG (Table 6).

**Table 5:** Gender-wise comparison of POAG cases and controls.

**Females with POAG vs. control females**				
Genotypg Genotypes	POAG females n = 85 (%)	Control females n = 172 (%)	p-value	OR(CI)
CC	55(64.70)	124(72.09)	0.471	
CT	29(34.11)	46(26.74)		
TT	1(1.17)	2(1.16)		
**Genetic Models**				
Dominant (CT+TT vs.CC)	30/55	48/124	0.249	1.41(0.80–2.46)
Recessive (TT vs. CT+CC)	1/84	2/170	0.992	1.01(0.09–11.3)
**Males with POAG vs. control males**				
Genotypes	POAG males n = 139(%)	Control males n = 195(%)	p-value	OR(CI)
CC	87(62.58)	137(70.25)	0.050*	
CT	52(37.41)	54(27.69)		
TT	0	4(2.05)		
Genetic Models				
Dominant (CT+TT vs. CC)	52/87	56/137	0.141	1.41(0.89–2.24)
Recessive (TT vs. CT+CC)	0/139	4/191	0.089	0.15(0.08–2.86)

* Indicates significant p-value

OR = Odds Ratio, CI = Confidence Interval

**Table 6:** The genotype distribution of -1562C>T MMP9 gene polymorphisms in patients with open-angle glaucoma (POAG) and angle closure glaucoma (PACG) according to CDR and IOP.

**POAG Cases**									
**Gene (rsID)** *MMP9* (rs3918242)	**Mean CDR**	**SD**	**Median**	**p-value**	**Size** **n = 224**	**Mean IOP**	**SD**	**Median**	**p-value**
**Genotype**									
CC	0.71	0.18	0.700	0.551	137	21.07	8.73	20.00	0.325
CT	0.77	0.86	0.700		85	22.45	8.40	20.00	
TT	0.75	0.21	0.750		2	19.00	9.89	19.00	
**PACG Cases**									
**Genotype**					**n = 138**				
CC	0.69	0.16	0.700	0.157	80	22.75	9.64	22.00	0.399
CT	0.74	0.12	0.700		53	23.96	7.56	24.00	
TT	0.78	0.08	0.800		5	23.20	11.07	19.00	

## Discussion

The present study investigated the association of rs3918242 (-1562C>T), a functional variant in the promoter region of *MMP9*gene with primary glaucoma in a north Indian population. In glaucoma pathogenesis, MMPs are important candidate genes because they can contribute to elevation in IOP by shifting the equilibrium between ECM synthesis and proteolysis within the TM, thereby modifying outflow resistance and hindering aqueous drainage [31]. Among different members of the MMP family, MMP9expression and activity has been extensively associated with glaucomatous optic neuropathies (GONs) [32]. The exact mechanistic details of how MMP9 contributes to GONs are not well understood but may involve detachment induced RGC death due to extensive ECM remodeling [32]. This has significant implication since the vision loss in glaucoma is due to irreversible damage to RGCs. That MMP9 may have a causal role in RGCs death was shown in a rat glaucoma model wherein the neuroprotection conferred by pyrrolidine dithiocarbamate (PDTC) to RGCs was mediated by downregulation of *Mmp9* [33]. Studies with *Mmp9* null mice also demonstrate altered TM composition, reduced aqueous humor drainage and IOP elevation [34] thereby establishing MMP9 as an important remodeler of TM. Apart from functional evidence, molecular genetics analyses in different populations have reported a link between the *MMP9*gene polymorphisms and POAG and PACG. In a Taiwanese population, highly significant association was observed between PACG and a non synonymous SNP in exon 6 of the *MMP9* gene (rs2664538; now merged into rs17576) [35]. Association with rs17576, along with another SNP (rs3918249) was replicated in an Australian Caucasian population [36], but not in Singaporean subjects [37] and neither in a Caucasian population from southern Austria [26]. A population based case control study on individuals of Chinese ethnicity, conducted by Shi et al revealed no significant association between the two variants of *MMP9* gene (rs17576 and rs3918249) and primary angle closure glaucoma [38]. Another study in Chinese Han population, investigated 4 SNPs (rs3918249, rs3918254, rs17577 and rs3787268) in *MMP9* gene, out of that only rs3918254 was found to be associated with PACG [39]. More recently, results of 2 meta-analyses seem contradictory in the sense that rs3918249 was affirmed to be associated with PACG by Rong et al [40] but not by Chen et al [41]. In the latter study, the authors in addition to performing a meta-analysis also evaluated common variants in *MMP9* for association with PACG which included rs3918249 in a Chinese population but did not get significant association with any of the 6 SNPs [41]. In yet another recent meta-analysis, *MMP9* rs17576 G > A polymorphism was observed to be a protective factor against the development of glaucoma [42]. However none of the above mentioned studies included promoter region polymorphisms in the *MMP9*, which therefore are not well investigated, both in POAG and PACG. rs3918242 is a C>T variant at position -1562 and affects the transcription of the gene; the CC genotype is associated with low promoter activity while CT, TT combinations exhibit higher activity [15]. The T substitution decreases the binding capacity of a putative transcription repressor protein, thereby causing an upregulation in the gene expression. In luciferase assays, MMP9 gene expression showed a 9-fold and 12-fold increase in the expression level for -1562 T/T genotype as compared to the -1562 C/T and -1562 C/C genotypes respectively [15]. Being a functional variant, it can act as a genetic risk factor for glaucoma development and progression. There are only two reports on the effect of -1562C>T variant on genetic predisposition to primary glaucoma, one in Caucasians from a Polish population [29] and the other is in a Pakistani population [43]. In our study, which is the first one to investigate *MMP9* polymorphism in any Indian glaucomatous population we got a significant association of rs3918242 with PACG under dominant as well as co-dominant model while marginal association was observed with POAG (p = 0.052). Our findings are consistent with the results of Markiewicz et al [29] in which a statistically significant association was obtained between rs3918242 and POAG. Stratification of the dataset on the basis of sex revealed higher risk towards PACG in males (p = 0.048, OR = 1.87; 1.00–3.05). Sex specific differences in genetic susceptibility to glaucoma could be accounted by the effect of estrogens on the levels of proinflammatory cytokines like IL-1beta which is known to induce *MMP9* expression in primary and immortalized cells [44]. In the Pakistani cohort of Punjabi origin, genetic association was observed between rs17576 and PACG but not with rs3918242 [43]. The contrasting results might be due to genetic admixture in the residing populations of respective areas (Punjab, India and Central Pakistan). The present study has some limitations. Only a single variant in the *MMP9* gene was studied. Assessing the role of other SNPs in the gene can allow for more powerful haplotype analysis to better elucidate the role of *MMP9* variants as a genetic risk factor for glaucoma. Due to lack of aqueous humor samples we were unable to correlate *MMP9* expression levels with the risk genotype and therefore there exists a possibility of presence of other functional variants in linkage disequilibrium with -1562C>T variant. Due to technical constraints, other endophenotypes of glaucoma viz retinal nerve fibre layer (RNFL), rim area (RA), axial length could not be correlated with -1562C>T which has been shown to influence these quantitative traits in POAG [36]. In conclusion, the study provides a first comprehensive data on a functional polymorphism (-1562C>T) in the *MMP9* gene in an Indian primary glaucomatous population. In our study population, the polymorphism was found to be associated with PACG and marginally with POAGwith CT genotype conferring a genetic risk for the condition. These results give additional impetus to investigate the role of *MMP9* gene polymorphisms in primary glaucoma in other populations and also to conduct functional studies to understand the role of *MMP9* in the pathology of POAG and PACG.
